# Empirical Evaluation of Single-Cell Foundation Models for Predicting Cancer Outcomes

**DOI:** 10.1101/2025.10.31.685892

**Published:** 2025-11-03

**Authors:** Haitham Elmarakeby, Ahmed Roman, Shreya Johri, Eliezer M. Van Allen

**Affiliations:** Dana Farber Cancer Institute, Boston, MA, USA

## Abstract

Foundation models pretrained on large-scale single-cell RNA sequencing data present a promising opportunity to advance translational cancer research. However, their utility in clinically relevant, patient-level applications of single-cell analysis remains underexplored. Here, we systematically evaluated nine emerging single-cell foundation models (scFMs) and three alternative baseline approaches across six cancer-specific tasks, ranging from subtype classification to treatment response prediction. We assessed model performance under zero-shot, continual training, and fine-tuning conditions, conducting 1,170 supervised and 130 unsupervised experiments. Our findings revealed that while current scFMs excel in certain analysis tasks, such as tumor microenvironment cell annotation, they had limited advantages in predicting clinical and biological outcomes of cancer patients compared to simpler baseline models. These insights highlight the critical role of evaluation on biologically and clinically relevant tasks in the responsible and impactful application of scFMs in precision oncology. They also emphasize the necessity of further methodological innovation and expanded cancer single-cell cohorts for the future development of scFMs.

## Introduction

Single-cell transcriptomic analysis of tumor tissues offers high-resolution insights into the cellular basis of cancer progression, therapeutic response, and microenvironmental dynamics ^1–3^. Inspired by advancements in natural language processing, foundation models pretrained on extensive single-cell datasets are increasingly applied to decode this complexity in non-cancer settings, and they may hold promise for interpreting cancer single cell data as well^4–7^. These models aim to internalize universal biological principles as “prior knowledge” ^8^, potentially improving tasks such as automated cell annotation to biomarker discovery. Initiatives such as CELLxGENE have facilitated these efforts by collecting and harmonizing the requisite large-scale data ^9^, enabling foundation models to represent cellular profiles analogously to how language models capture structure and meaning in natural language ^5–7,10^.

Despite their potential, an essential question is whether the embedded prior knowledge in these pretrained models translates into demonstrable advantages for cancer-specific problems, including predicting patient-level clinical and biological outcomes. While existing evaluations have focused on measuring foundation models’ performance in predicting cell-level outcomes ^11–16^, whether these models can meaningfully extend from cell-level patterns to patient-level clinical or biological states that are crucial for translational impact (e.g. prognostication, therapy resistance)has not yet been thoroughly tested. This gap is especially pronounced in the cancer domain, where transcriptional heterogeneity and microenvironmental context play pivotal roles in shaping disease progression and treatment response. Yet, evaluating the utility of the foundation models in informing our understanding of the transcriptional basis of clinical and biological outcomes of cancer patients is essential for advancing precision oncology and guiding therapeutic strategies. Moreover, understanding the factors that influence foundation model performance will not only help researchers select optimal analytical strategies but also illuminate principles that can guide the design of next-generation models capable of deeper biological reasoning and broader translational impact.

**Figure 1|:**
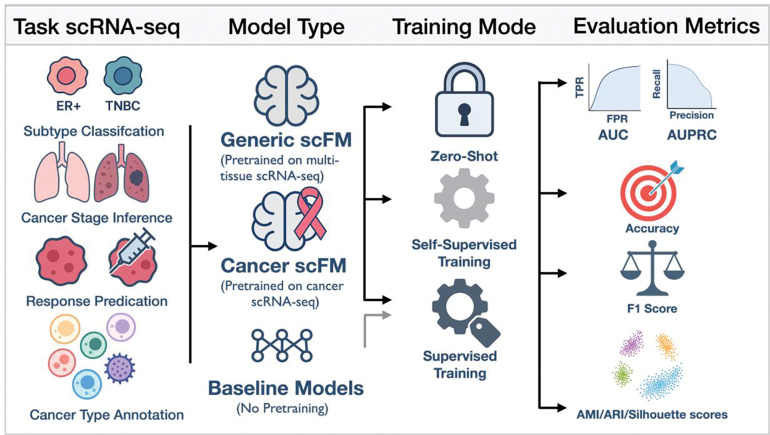
Overview of our benchmarking framework. Schematic of the end-to-end evaluation pipeline. From left to right: Tasks scRNAseq: We challenge each model on four representative cancer-single-cell classification problems—subtype discrimination (ER^+^ vs. TNBC), cancer stage inference, therapeutic response prediction, and tissue-of-origin annotation. Model Type: We compare two single-cell foundation models—Geneformer (pretrained on multi-tissue or cancer-specific corpora) and scGPT (general vs. pan-cancer)—against three “no pretraining” baselines (highly variable gene selection, PCA, and scVI). Training paradigms: Zero-shot: freeze the pretrained embedding extractor, train only a downstream Random Forest classifier; Continual (self-supervised) pretraining: further pretrain the foundation model on the unlabelled training split; Supervised fine-tuning: train the entire model end-to-end on labelled data. Evaluation metrics: Supervised: classification AUC, AUPRC, accuracy, F1_1_, precision, and recall; Unsupervised: embedding quality via ARI, NMI, average silhouette width. This unified schematic highlights how each model and training regimen is tested under exactly the same data split, feature input, and evaluation conditions across all cancer tasks.

Here, we present an extensive and systematic analysis of the performance of leading scFMs pretrained on large-scale datasets relevant to cancer research (Figure 1). In addition to tumor microenvironment cell type annotation, our evaluation explored the performance of scFMs compared to multiple baseline approaches in six different translational oncology tasks using a variety of strategies and paradigms. In total, we conducted 1,170 supervised prediction tasks (234 experiments, each repeated five times with different data splits) and 130 unsupervised experiments (13 experiments, each repeated ten times) to benchmark the tested scFM models against three baseline approaches, providing a thorough assessment of their capabilities. Through this systematic evaluation, we determined that existing foundation models require further innovations in methodology and cohort sizes to advance cancer-specific investigations. Ultimately, our findings highlight that without domain-specific adaptations and rigorous benchmarking, current scFMs risk underperforming in clinically important oncology applications and thus demand careful methodological refinement before translational deployment.

## Methods

### Foundation Models:

We benchmarked twelve models, including nine single-cell foundation models (scFMs) and three baseline architectures, across six representative cancer single-cell classification tasks (Figure 1).

The scFMs were pretrained on datasets consisting of 11M to 104M single-cell profiles and ranged from 10M to 316M parameters (Table 1). The tasks spanned subtype discrimination (estrogen receptor positive [ER^+^] vs. triple negative breast cancer [TNBC]), cancer stage inference, therapeutic response prediction, immune exhaustion status, and tissue-of-origin annotation. Among the scFMs, four were Geneformer ^5^ variants encompassing the first generation (GF-V1)^5^ and second generation (GF-V2)^17^ models. A cancer-specific version of GF-V2 (GF-V2 [cancer]) finetuned on cancer cells was also evaluated. Two scGPT models were included, representing general-purpose and cancer-specific variants. The remaining scFMs - scFoundation ^7^, SCimilarity ^18^, and CellPLM ^19^, extended the comparison to models differing in architecture and pretraining objectives (Table 1).

To benchmark scFMs, we compared them with three baseline methods under matched experimental conditions, each reflecting a method of dimensionally reducing single-cell data representation. First, the Highly Variable Genes (HVG) baseline^20,21^ represents explicit feature selection. The top 4096 highly variable genes were selected from the expression matrix to serve as direct input features, matching the dimensionality of the evaluated scFMs. Second, the Principal Component Analysis (PCA) baseline represents a standard feature selection based on variance explained. The top 100 principal components were extracted from the transcriptional expression matrix. Third, the single-cell scvi-tools variational autoencoder (scVI) ^22^ baseline represents non-linear probabilistic modeling of gene-gene dependencies. Unsupervised training of a 2-layer variational autoencoder was performed on 4098 HVGs with a negative binomial gene-likelihood, and the extracted 100 dimensional latent embeddings were used for the downstream analysis. For supervised tasks, both scFMs and baseline models were combined with a random forest with 100 trees and a maximum depth of 5 to predict the desired outcomes.

Our benchmarking analysis evaluated single-cell foundation models (scFMs) across three distinct training paradigms on multiple cancer datasets. In the zero-shot paradigm, scFMs generated embeddings for both training and test datasets without any task-specific supervision; these embeddings were evaluated for compactness of the embedding and the alignment of the clustering with clinical or biological status. For supervised prediction tasks, the cell embeddings were combined with a random forest model to generate predictions. In the task-specific continual training paradigm, scFMs were first updated by continually training them using the task-specific training data without labels (unsupervised domain adaptation), after which the resulting embeddings were compared to the zero-shot embeddings. In the fine-tuning paradigm, the entire scFM was trained end-to-end with labeled training data, jointly optimizing both embedding extraction and classification head, and evaluated directly on the test data.

We evaluated the performance of the tested models using six different cancer-specific tasks, including supervised and unsupervised tasks. All models were evaluated using their ability to dissect the tumor microenvironment and identify clusters corresponding to different cell types without prior training (zero-shot). The resulting embeddings from each model were clustered and evaluated using a set of metrics to assess the alignment with known cell types. We applied a set of unsupervised clustering metrics, including adjusted Rand index (ARI) and normalized mutual information (NMI) between predicted clusters and known labels, and average silhouette width (ASW). The metrics were calculated for 10 different subsamples bootstrapped from the whole dataset to study the stability of the metrics.

Five different supervised tasks were performed on each model to measure the performance of the tested models regarding predicting patient-level outcomes. These tasks involved (i) the prediction of cancer subtypes in breast cancer; (ii) identifying treatment effects, including target therapy, immune therapy, and chemotherapy in breast and lung cancer; and (iii) predicting immune cell exhaustion status. The embedding from each model was extracted, and a classifier (random forest or neural network) was trained on the frozen embedding to predict the corresponding outcome. In the case of end-to-end fine-tuning of the scFMs, a neural network classifier was added to the model, and both the information extraction component and the classifier component were trained jointly. Supervised classification was evaluated using AUC, AUPRC, F1-score, accuracy, precision, and recall, and these metrics were evaluated for five different cross-validation folds. Details of tasks, models, and data are provided in Supplementary Table 1.

To obtain patient-level predictions from cell-level embeddings, we compared three distinct aggregation strategies. In the majority-vote approach, cell-level predictions were independently generated by the trained classifier, and the most frequent prediction determined the patient-level label. In the pseudo-bulk averaging approach, cell embeddings were averaged to produce a single patient-level representation, which was subsequently classified using an RF classifier. Finally, in the multiple-instance learning (MIL) approach, an attention-based neural network directly learned patient-level predictions by weighting individual cell embeddings. Each model was systematically evaluated across multiple cancer datasets using the three training paradigms and aggregation strategies, providing a thorough assessment of predictive performance and robustness.

## Results

### scFMs Yield Superior Unsupervised Embeddings for Tumor Microenvironment Annotation

We first evaluated the capacity of single-cell foundation models (scFMs) to generate embeddings that capture biologically relevant structure in an unsupervised setting. We evaluated four different Geneformer models, including Geneformer version 1, and 2 (GF-V1, GF-V2), a cancer-specific version (GF-V2 [cancer]), and a deep version (GF-V2 [deep]). For GF-V1, we also evaluated the effect of task-specific adaptation by continually training the model without labels, denoted as GF-V1 [continue] . We also tested two versions of the scGPT family, including a generic version (scGPT) and a cancer-specific version (scGPT [cancer]). In addition, we tested three other scFMs, including scFoundation, SCimilarity, and CellPLM. Finally, we included three different baseline models; highly variable genes (HVG), principal component analysis (PCA), and single-cell variational autoencoder (scVI) (see Table 1, Supplementary Table 1, and Methods).

**Figure 2 |:**
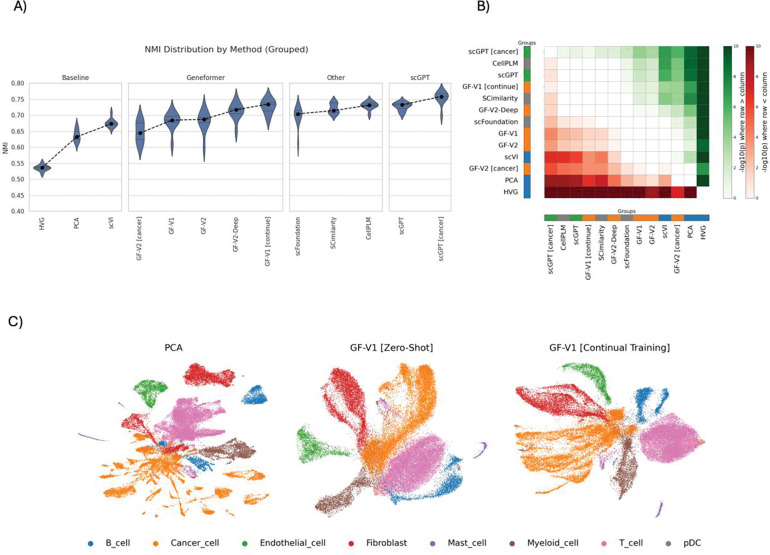
Performance of different models on annotating the tumor microenvironment. A) A comparison of the Normalized Mutual Information (NMI) calculated between clusters of the embedding space and the different microenvironment cell types. The embeddings of each model are subsampled by replacement 10 times, and the NMI score for each run is calculated. In general, foundation models achieve better clustering scores with a continually trained model based on Geneformer V1 (Gf-V1 [continue]), achieving the best performance among Geneformer models. B) Statistical test between the performance of all models for the tumor microenvironment cell annotation task. The intensity of each cell corresponds to −log(p) of the test between rows and columns (green (red) *i*, *j* element of intensity map means model *i* is statistically overperforms (underperforms) model *j*). The color indicates the directionality of the test. scGPT [cancer] shows a significant increase over all other models. C) UMAP plots illustrating cell-type separability across representative methods: scVI (left), GF-V1 [Zero-Shot] (middle), and GF-V1 [Continual Training] (right). GF-V1 embeddings after continual training show markedly improved intra-cluster compactness and more precise inter-cluster boundaries in agreement with quantitative clustering metrics reported in panel A, highlighting the benefits of domain-adaptive continual pretraining.

Using a tumor microenvironment cell annotation task with a cohort of profiled solid tumors (n = 31 tumors and ~41K cells) with ground truth labels ^23^, we assessed the quality of embeddings by computing the normalized mutual information (NMI) between the unsupervised clusters of the embedding space and known cell-type labels (Figure 2A; additional clustering metrics, including adjusted Rand index (ARI) and silhouette width (ASW), are reported in Supplementary Figure 1). The first and second-generation Geneformer models achieved moderate performance, exceeding that of the baseline models but remaining below the larger GF-V2-Deep model. Among all Geneformer variants, the continually trained GF-V1 [continue] model achieved the highest NMI score, reflecting the strongest concordance between learned embeddings and underlying biological cell types. These results suggest that continual training can enhance the representational capacity of smaller models, enabling them to substantially outperform larger counterparts on cell annotation tasks. Variants of scGPT models demonstrated overall superior performance, with the scGPT [cancer] variant achieving the best results among all evaluated models while maintaining low variability. Of note, CellPLM exhibited comparable performance relative to the scGPT model variants despite its smaller training corpus. The weakest performance was observed for HVG, which fell below all other models.

To further quantify these differences, we performed statistical comparisons between models using paired statistical tests (Figure 2B). scGPT [cancer] embeddings showed a significant increase in NMI over all other approaches, except scGPT and CellPLM, which performed comparably. Among the Genformer models, GF-V1 [continue] exhibited significantly improved performance compared to all other Genformer models with the exception of GF-V2-Deep (Figure 2B and Supp Table 4]). These statistical tests reinforced the importance of continual pretraining for improving unsupervised embedding quality, even relative to deeper or larger models. Moreover, this result highlights the role of domain adaptation in improving performance in large models.

In addition to the quantitative metrics, UMAP visualizations provided a qualitative perspective on how embedding quality might relate to underlying cellular structure, (Figure 2C). Compared to PCA, which appeared to produce more fragmented and overlapping clusters, GF-V1 [Zero-Shot] improved the perceived separation of major cell types. After continual training, GF-V1 [continue] embeddings exhibited tighter, and visually distinct clusters aligned with annotated cell types and potentially reflecting finer intra-cluster differences less evident in other embeddings (see supplementary Figure 2 for a quantitative account of cluster and cell type marker alignment). While these observations are qualitative and exploratory in nature, they may reflect the improvements captured by NMI and suggest that continual adaptation could enhance the biological relevance of the learned embedding space

Together, these results highlight the benefit of continual pretraining on domain-specific data for generating high-quality, biologically relevant representations. While scaling model size and pretraining corpus does improve the quality of embeddings for tumor microenvironment annotation, continual training on task-specific data provided an impactful way to enhance the performance of smaller models. This finding highlights that thoughtful model development and training strategies, e.g. through continual training, can outweigh the benefits of merely increasing dataset size or model scale, offering a more practical and efficient approach for capturing fine-grained biological structure in disease specific contexts like cancer.

### Evaluating the Predictive Performance of scFMs in Translational Oncology Domains

Next, to systematically evaluate the supervised predictive performance of scFMs, we assessed them across a diverse set of clinically relevant classification tasks derived from lung adenocarcinoma (LUAD) and breast cancer (BRCA) cohorts. In each task, the goal was to predict patient-level clinical outcomes using aggregated information from single-cell profiles. These tasks were designed to capture distinct biological contrasts and span a spectrum of prediction difficulties, thus providing a systematic benchmarking framework. Specifically, we included: (i) distinguishing treatment-naïve from tyrosine kinase inhibitor (TKI)-treated patients using cancer cells; (ii) differentiating treatment-naïve from anti-PD1–treated BRCA patients using T-cells; (iii) classifying estrogen receptor positive (ER+) versus triple-negative breast cancer (TNBC) patients based on cancer cells; (iv) differentiating exhausted versus non-exhausted T-cell states in BRCA tumors; (v) distinguishing treatment-naïve from neoadjuvant chemotherapy–treated BRCA patients using cancer cells; and (vi) classifying early-stage versus late-stage LUAD. (We note that the early-stage versus late-stage LUAD task exhibited particularly high variability in performance metrics (i.e., a low signal-to-noise ratio as reflected by mean-to-standard deviation ratios), which made robust comparison difficult and are reported in Supplementary Figure 4).

Then, we pursued a systematic evaluation of scFMs for each of these cancer outcome predictions across multiple analytical dimensions: evaluating pseudo-bulk performance, quantifying the effects of model scale and cancer-specific adaptation, comparing patient-level aggregation strategies, assessing gains from end-to-end fine-tuning over frozen embeddings, and anchoring evaluation in AUC/AUPRC as robust cross-task metrics.

#### Evaluating scFM Performance Under Pseudo-Bulk Aggregation

**Figure 3 |:**
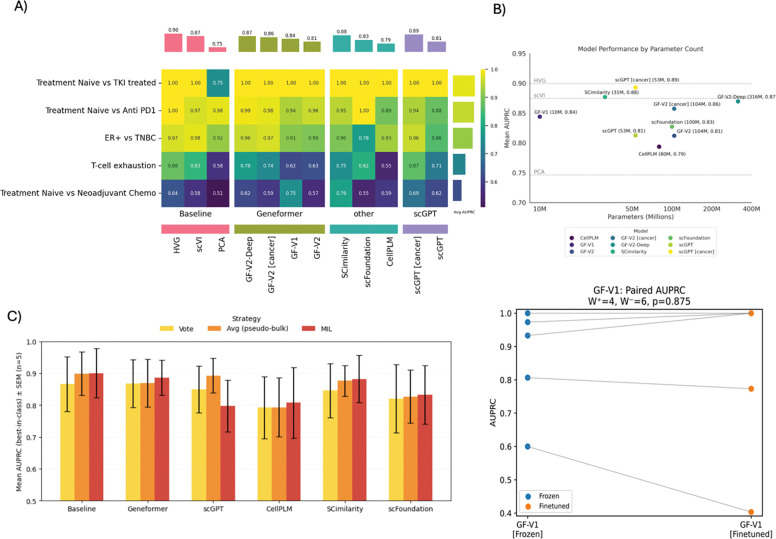
Performance of different models on predicting cancer outcomes. A) Comparison between all models in predicting five different outcomes measured using the area under the precision-recall curve, AUPRC. Each cell represents the average of five cross-validation experiments. The bars on top show the average performance of each model across the different tasks. B) The relationship between model performance (AUPRC on y axis) and size (# params in million on x axis). C) Comparison between different classification schemes used for each model (Avg= average expression/embedding, Vote= majority vote of cells in each patient, MIL= Multi-Instance Learning) D) End-to-end fine-tuning vs building a classifier on top of frozen embeddings show no statistically significant difference with a *p*-value =0.875.

We first examined patient-level prediction performance under the pseudo-bulk aggregation strategy (other strategies are presented in Supplementary Figure 3), a common approach for summarizing all cells from each patient into a single embedding vector before classification. Under the pseudo-bulk strategy - where all cell embeddings from a patient are averaged into a single embedding vector before classification - performance depended less on the accuracy of individual cell predictions and more on how well important biological differences were preserved in the averaged embedding (Figure 3A).

In this regime, the cancer-adapted scGPT (scGPT [cancer]) achieved the highest mean AUPRC (~0.89 ± 0.11), closely followed by SCimilarity (~0.88 ± 0.10), GF-V2-Deep (~0.87 ± 0.15). A similar pattern held within the Geneformer foundation model family: GF-V2 [cancer] (~0.86 ± 0.16) notably outperformed its generic counterpart GF-V2 (0.81 ± 0.18), indicating that cancer-specific pre-training remains beneficial even after strong averaging and may improve patient-level signal.

Among baseline methods, HVG remained competitive when biological contrasts were strong, achieving a high overall mean (~0.90 ± 0.15) and slightly outperforming the best foundation models on average in these classification tasks. scVI (0.87 ± 0.16) was comparable in average performance to the best foundation models while PCA (0.75 ± 0.18) had larger performance drops compared to simply selecting the highly variable genes or using scVI. On the most challenging task (determining whether the tumor was treatment-naïve or had been exposed to neoadjuvant chemotherapy), the highest single-task AUPRC was achieved by the generic SCimilarity (0.79).

#### Impact of Model Size and Cancer-Adaptation on scFM Performance

To evaluate how model size and cancer-specific adaptation affect predictive performance across cancer tasks, we asked whether larger parameter counts or cancer-specific pretraining improve mean AUPRC. While models with more parameters generally achieved higher mean AUPRC, parameter count alone did not guarantee superior performance (Figure 3B). For instance, the 316M-parameter GF-V2-Deep, one of the largest models examined herein, ranked among the highest in the AUPRC across all tasks (≈0.87); however, smaller cancer-adapted models - such as GF-V2 [cancer] (104M) and scGPT [cancer] (53M) - matched or even slightly surpassed its performance, with mean AUPRCs of 0.86 and 0.89 respectively. SCimilarity (31M), a tenth of the size of GF-V2-Deep (316M) and trained on a much smaller data corpus, outperformed GF-V2-Deep without cancer-specific training. Conversely, their generic counterparts, GF-V2 (104M) and base scGPT (53M), trailed their size-matched, cancer-adapted versions, further underscoring the added benefit of domain-specific training. Although CellPLM excelled in cell-type annotation, it underperformed all of its counterpart scFMs in the benchmarking classification tasks, highlighting performance task dependence and potentially the need for hyperparameter tweaking to get better performance. Furthermore, the relatively parameter-free HVG baseline remained competitive with several foundation models. Thus, sheer model size was insufficient in these use cases; instead, cancer adaptation yielded substantial performance gains with the most efficient parameter utilization.

#### Comparing Aggregation Strategies for Patient-Level Predictions

We next proposed and evaluated multiple aggregation strategies to generate patient-level predictions. Since all tested foundation models can generate cell-level predictions, we devised three different strategies to convert cell-level predictions/embeddings to patient-level predictions: i) majority-vote, ii) pseudo-bulk averaging, and iii) multiple-instance learning (MIL). We observed that the choice of cell-to-patient aggregation strategy can shift AUPRC by as much as or more than the choice of embedding model itself (Figure 3C). For the baseline methods (HVG, PCA, scVI), a Multiple-Instance Learning (MIL) attention head, which learns to weight individual cell contributions, yielded mean AUPRC (0.90 ± 0.07), performing similarly to pseudo-bulk averaging (Avg) (0.90 ± 0.07; where cell embeddings are averaged into a single patient representation). Both strategies outperformed majority vote (Vote) (0.87 ± 0.08; where each cell “votes” and the most frequent prediction becomes the patient-level call; see Supplementary Figure 3). Within the Geneformer family, MIL (0.89 ± 0.06) outperformed pseudo-bulk averaging (0.87 ± 0.07) and majority vote (0.87 ± 0.07). For the scGPT family, averaging (0.89 ± 0.05) outperformed voting (0.85 ± 0.07), and both exceeded MIL (0.80 ± 0.08). Among aggregation strategies, MIL achieved the highest mean AUPRC (0.866 ± 0.041), pseudo-bulk averaging reached comparable though slightly lower performance (0.844 ± 0.045), and majority vote exhibited the lowest variability (0.842 ± 0.023), highlighting a trade-off between absolute performance and consistency across tasks. These results underscore that aggregation strategy, whether attention-based MIL, majority-vote, or pseudo-bulk average, is an important design consideration for patient-level prediction in cancer settings and comparable to the choice of embedding model.

#### Evaluating the Impact of End-to-End Fine-Tuning on scFM Performance

We then examined the performance changes achieved by end-to-end fine-tuning of scFMs for classification in comparison to frozen embeddings (Figure 3C). On average, the fine-tuned GF-V1 model exhibited a similar AUPRC compared to its frozen counterpart (where embeddings were fixed and only a separate classifier was trained), highlighting the challenge of training large models on a small number of patient samples. Finetuning GF-V1 performance was not consistent across tasks: while three out of five tasks showed gains, two tasks exhibited declines. The Wilcoxon signed-rank test likewise yielded a non-significant result (p ≈ 0.88), reflecting variability in task-level changes. Crucially, despite these trends, the mean AUPRC of the fine-tuned model remained comparable to that of the top-performing baseline methods (such as HVG under pseudo-bulk averaging), rather than decisively surpassing them. This finding was further supported by a wide 95% bootstrap confidence interval (−0.11 to +0.04), a non-significant p-value (≈ 0.88 for GF-V1 [finetune]), and a similarly non-significant p-value (≈1.00) for GF-V2 [finetune] (Supplementary Figure 5). Together, these findings suggest that while end-to-end fine-tuning in this undersampled regime (which characterizes cancer molecular profiling datasets more generally) may not provide a discernible gain, its benefits did not consistently outweigh strong baseline strategies when evaluated across multiple, diverse clinical oncology tasks.

#### Metrics for Cross-Task Performance Evaluation

**Figure 4 |:**
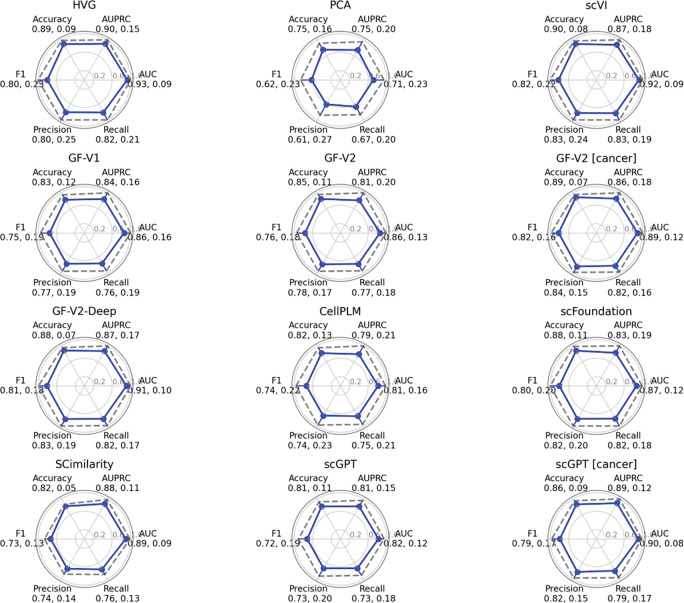
Evaluation of cross-task performance The figure comprises twelve separate radar charts—arranged in a 4 × 3 grid—corresponding to the embedding methods HVG, PCA, scVI, scGPT, scGPT [cancer], GF-V1, GF-V2, GF-V2 [cancer], GF-V2-Deep, CellPLM, scFoundation, and SCimilarity. Each chart has six equally spaced radial axes representing, in clockwise order, Accuracy, AUPRC, AUC, F1, Recall, and Precision. An outer circle marks the unit radius (score = 1) and a corresponding dashed gray polygon indicates the standard deviation. For every model, a solid blue polygon connects the mean score of all classification tasks on each metric, and a numeric value pair of the mean score (left) and its standard deviation (right) are written below the score name at the end of each axis. **All models in this graph are trained using an averaging approach to generate patient-level outcomes**. Other aggregating schemes are shown in Supplementary Figures 5 and 6.

Finally, we performed a multi-metric evaluation to identify metrics that support reliable cross-task comparisons under pseudo-bulk aggregation in Fig. 4 (with MIL and vote in Supplementary Figs. 5–6). Because oncology datasets are small and often imbalanced, we treat AUPRC as the primary endpoint and report AUC alongside it. Across all models, the average of AUC and AUPRC was 0.875 and 0.853, respectively. In terms of stability, accuracy (average SD ≈ 0.10) and AUC (≈ 0.12) showed the smallest dispersion across tasks, whereas AUPRC (≈ 0.17) and thresholded metrics—F1, precision, recall (≈ 0.18–0.20)—were more variable. For example, GF-V2 [cancer] achieved AUC ≈ 0.89 ± 0.12 and AUPRC ≈ 0.86 ± 0.18. Notably, the HVG baseline remained a strong reference (AUC ~0.93 ± 0.09; AUPRC ~0.90 ± 0.15), while the larger general foundation models and cancer-specific foundation models (e.g., GF-V2-Deep, scVI, scGPT [cancer]) performed comparably (albeit slightly lower) on AUC/AUPRC.

## Discussion

Our benchmarking study systematically explored the capabilities of single-cell foundation models (scFMs) across multiple dimensions: embedding quality, patient-level prediction performance, model size, domain adaptation, aggregation strategies, and metric reliability. Collectively, these analyses revealed that while scaling model size generally improves performance, domain-specific training and task-specific continual training offered an efficient and effective path to improved outcomes. For example, domain-adapted models, such as GF-V2 [cancer] and scGPT [cancer], frequently matched or surpassed larger models like GF-V2-Deep, highlighting that targeted adaptation can provide meaningful gains without relying solely on parameter scale. This finding underscores the importance of leveraging task-specific data and continual training strategies to fully realize the potential of scFMs in clinical applications.

In patient-level outcome prediction, our analysis demonstrated the important influence of cell-to-patient aggregation strategies on the ultimate performance of the tested models. We showed that multi-instance learning (MIL) generally led to the highest performance among models except scGPT, emphasizing the benefit of devising an adaptive method of combining cell-level predictions. While MIL had the highest performance, it also had the largest variability. Pseudo-bulk averaging reached comparable though slightly lower performance, and the majority vote exhibited the lowest predictive performance but also a lower variability. This suggests that the aggregation strategy can influence predictive performance as much as, or even more than, the choice of embedding model itself, warranting careful consideration in future work.

Of note, this study has several important limitations. First, our benchmarks focused on cancer-related tasks using existing single-cell datasets, where the number of available patient samples is typically small, especially when focusing on clinically relevant outcomes where each patient, rather than each cell, serves as the effective sample. This limited sample size, typically of the order of a few tens of patients or less, constrains the ability of scFMs to fully leverage transfer learning and may bias performance estimates, potentially leading to unreliable estimation of their performance. In addition, while we systematically evaluated multiple aggregation strategies and domain adaptations, we did not exhaustively explore more sophisticated strategies for representing per-patient cell-cell interactions.

In addition, neural network models could benefit from hyperparameter tuning, adaptive learning rate schedules, and early stopping. These techniques were intentionally excluded to maintain a controlled comparison space. Moreover, threshold-based metrics, including Accuracy, Precision, Recall, and F1 were calculated using a default threshold of 0.5. Using adaptive methods to calculate the threshold can lead to different results. It is worth noting that all the comparisons in this study are based solely on single-cell transcriptional data, without accounting for other data modalities inferrable from single cell analysis (e.g. copy number information, an essential dimension of tumor biology that captures genomic alterations driving cancer progression and treatment response). Finally, our reliance on pseudo-bulk and other standard aggregation schemes may overlook alternative biologically informed approaches to integrating cell-level signals that could better account for patient heterogeneity and improve outcome prediction by revealing underlying disease mechanisms.

Broadly, our work underscores the significant methodological and conceptual challenges that continue to constrain the development of single-cell foundation models (scFMs). Current scFMs typically rely on simplified representations of cells as ranked or ordered lists of genes, overlooking the rich network of relationships between genes and higher-order biological entities such as pathways and cellular processes. This abstraction limits the models’ ability to capture the multiscale organization inherent in biological systems generally, and cancers in particular. Moreover, most scFMs treat cells as independent observations, failing to account for critical aspects of cell–cell communication and tissue-level organization that are often dysregulated in oncogenesis. Another major limitation lies in the lack of biological and clinical context: existing models often disregard the influence of tissue type, disease state, and microenvironmental setting on cellular behavior. Similarly, they lack sample-level representations that are essential for translational and clinical applications, such as predicting patient outcomes or treatment responses.

Simply expanding dataset size alone is unlikely to overcome these limitations. Nonetheless, it remains essential to collect samples from multiple patients across a diverse range of cancer types, while ensuring that foundation models explicitly integrate the full tumor microenvironment to capture intercellular interactions and contextual biological signals. Future progress will require careful attention to clinical and biological context, improved feature engineering, and the incorporation of structured prior knowledge that reflects biological organization and constraints. Advances in loss function design and representation learning will also be necessary to develop models that can reason in biological terms and provide interpretable, mechanistically grounded insights for downstream tasks in cancer research and beyond.

## Conclusion

In this benchmarking study, we systematically evaluated single-cell foundation models (scFMs) across an extensive set of tasks, revealing several important insights for their use in cancer research and clinical outcome prediction. We first demonstrated that scFMs produce improved unsupervised embeddings for tumor microenvironment annotation, particularly when leveraging continual domain adaptation, thereby facilitating clearer delineation of complex cellular landscapes. Moving into supervised patient-level prediction tasks, we showed that pseudo-bulk aggregation effectively preserves task-relevant variation, enabling domain-tuned foundation models (e.g. GF-V2 [cancer] and scGPT [cancer]) to achieve comparable or better performance than larger models, without requiring excessively large parameter counts.

Further, we found that while model scaling contributes to improved performance, it is the combination of domain-specific continual pretraining and thoughtful aggregation strategy that yields meaningful gains, underscoring that bigger models alone are not sufficient. Our analysis of aggregation strategies revealed that the choice of how to integrate cell-level information into patient-level predictions can influence performance as much as, or more than, the choice of embedding model itself. Moreover, end-to-end fine-tuning, though providing task-specific improvements in certain settings, did not consistently surpass strong baseline approaches, highlighting the importance of careful model adaptation and validation.

Looking ahead, integrating domain-adapted scFMs with explicit biological priors and multi-modal data may provide a route to more interpretable, data-efficient, and clinically relevant models. We anticipate that these insights and recommendations will guide the next generation of single-cell modeling efforts, helping to bridge the gap between foundational embeddings and real-world translational applications.

## Supplementary Material

Supplement 1

## Figures and Tables

**Table 1 |:** Summary of foundation and baseline models evaluated. Geneformer V1 and V2 differ in parameter count, depth, and input size, with V2 including deeper and cancer-adapted variants. scGPT variants share architecture but include a cancer-tuned version. scFoundation, CellPLM, and SCimilarity are trained on normal cells with no cancer-specific variants, while CellPLM included 2M Spatial Resolved Transcriptomics (SRT). Baseline methods (HVG, scVI, PCA) use 2048 genes without pretraining.

Model	Corpus size	Params	Depth	Input size

**Geneformer VI**				
GF-V1	~30M	10M	6	2048
**Geneformer V2**				
GF-V2	~104M	104M	12	4096
GF-V2 [cancer]	~104M + 14M cancer	104M	12	4096
GF-V2-Deep	~104M	316M	20	4096

**scGPT**				
scGPT	~33M	53M	12	2048
scGPT (cancer)	~33M + 5.7M cancer	53M	12	2048

**Other foundation models**				
scFoundation	> 50M	100M	not reported	19,264
CellPLM	~9M scRNA + 2M SRT	82.4M	4	~10^4^ (cells)
SCimilarity	23.4M	31M	4	28,231

**Baselines**				
HVG	–	–	–	4096
scVI	–	–	–	4096
PCA	–	–	–	4096

## Data Availability

Raw data can be downloaded from the original publication as listed in the Supplementary Information. The Derived data is deposited in this link Code is available on github/marakeby/scFM_eval
