# Supplementary material for: Empirical Evaluation of Single-Cell Foundation Models for Predicting Cancer Outcomes
Source: bioRxiv. 2025 Nov 3:2025.10.31.685892. Preprint. [Version 1] doi: 10.1101/2025.10.31.685892 (PMC12637420; doi:10.1101/2025.10.31.685892)
Supplement: Supplement 1 [file NIHPP2025.10.31.685892v1-supplement-1.pdf]

bioRxiv preprint doi: https://doi.org/10.1101/2025.10.31.685892; this version posted November 3, 2025. The copyright holder for this preprint (which was not certified by peer review) is the author/funder, who has granted bioRxiv a license to display the preprint in perpetuity. It is made available under aCC-BY 4.0 International license.

Supplementary Information

Empirical Evaluation of Single-Cell Foundation Models for Predicting Cancer Outcomes

Data

We used four publicly available datasets, spanning two cancer types and six comparison groups, for our analysis -
(i) Bassez et al. 2021 (breast cancer, ER+ and TNBC)
(ii) Kim et al. 2020 (lung adenocarcinoma),
(iii) Qian et al. 2020 (lung adenocarcinoma)
(iv) Maynard et al. 2020 (lung adenocarcinoma)

Bassez et al. 2021 [1]:
We downloaded raw data (.rds objects and .csv metadata files) from https://lambrechtslab.sites.vib.be/en/single-cell. The dataset contained ER+, HER2+ and TNBC patients who were treatment-naive, anti-PD1 treated or chemotherapy + anti-PD1 treated. We removed HER2+ patients from our analysis because they were few in number. We then analyzed the dataset in four supervised ways -

(i) Treatment-naive ER+ vs. treatment-naive TNBC,
(ii) Treatment-naive vs. anti-PD1 treated,
(iii) T-cell exhausted vs. non exhausted, and
(iv) Treatment-naive vs. neoadjuvant chemo treated.

In addition to the supervised tasks we tested all the models for their capacity to dissect cell types in an unsupervised way. Thai included cancer cells, T-cells, B-Cells, Author annotations for tumor cells and T-cells in addition to other cell types were used.

Kim et al. 2020 [2]:
We downloaded raw counts (GSE131907_Lung_Cancer_raw_UMI_matrix.txt.gz) and cell-type annotations (GSE131907_Lung_Cancer_cell_annotation.txt.gz) from GSE131907. Additional metadata about patient history was downloaded from Table S1 present in the paper. Only patient samples from the primary tumor site were retained for further analysis, and samples from pleural effusions and metastasis sites were removed. Author annotations for tumor cells were used.

bioRxiv preprint doi: https://doi.org/10.1101/2025.10.31.685892; this version posted November 3, 2025. The copyright holder for this preprint (which was not certified by peer review) is the author/funder, who has granted bioRxiv a license to display the preprint in perpetuity. It is made available under aCC-BY 4.0 International license.

Maynard et al 2020 [3]:
We downloaded raw counts from https://drive.google.com/drive/folders/1qZJiFFf9ggfi0Sn79n8uOhWqVDfvjKHp. NI01_Nonimmune_Seurat_object_annotated.RData contained all epithelial cells, and NI05_all_epithelial_annotated_normal_and_tumor.RData contained annotations for tumor vs. non-tumor cells. Additional metadata was downloaded from Supplementary Table S1 of the paper. Only samples acquired from primary tumor sites, and those from patients diagnosed with lung adenocarcinoma were retained. Other samples were removed from future analysis.

Qian et al 2020 [4]:
We downloaded raw data for lung adenocarcinoma from https://lambrechtslab.sites.vib.be/en/pan-cancer-blueprint-tumour-microenvironment-0. Author annotations for tumor cells were used. Additional metadata information (tumor stage) was acquired from Supplementary Table S1 of the paper.

Meta-analysis of Lung Adenocarcinoma:
Data from the three lung cancer datasets was jointly analyzed - (i) early-stage treatment-naive vs. late-stage treatment-naive patients, (ii) late-stage treatment-naive vs. late-stage TKI-treated patients. We classified stages I, II and IIIA as early-stage tumors, and stage IV as late-stage tumors, consistent with Kim et al. 2020. Patients with stage IIIB were excluded from analysis.

bioRxiv preprint doi: https://doi.org/10.1101/2025.10.31.685892; this version posted November 3, 2025. The copyright holder for this preprint (which was not certified by peer review) is the author/funder, who has granted bioRxiv a license to display the preprint in perpetuity. It is made available under aCC-BY 4.0 International license.

Methods

We developed a modular machine learning pipeline to streamline the analysis of high-dimensional datasets, particularly single-cell data. The workflow is configurable via a YAML specification and composed of several stages: data loading, quality control, preprocessing, feature extraction, model training, evaluation, and visualization. The pipeline is implemented in Python and leverages standard machine learning and data science libraries.

Quality Control and Preprocessing

Quality control (QC) steps are configurable and may include filtering cells or genes based on customizable thresholds (e.g., minimum gene counts or cell coverage). Preprocessing typically involves normalization using standard methods such as z-score scaling

Feature Extraction and Embedding

Feature extraction is performed using dimensionality reduction techniques. For visualization and downstream modeling, embeddings can be computed using Principal Component Analysis (PCA) or other methods as defined in the configuration. The resulting low-dimensional embeddings are used for both visualization and classification.

Classification and Evaluation

A classifier (e.g., Random Forest) is trained using the extracted embeddings as input features and the label annotations as targets. The classifier is evaluated on prediction accuracy and other metrics including AUC, AUPRC, F1, precision, and recall.

Configuration and Execution

Each experiment is defined in a YAML file that specifies the modules and parameters for each pipeline stage. The pipeline dynamically loads the required classes and executes each step in sequence. Logging is enabled for all steps, with log files named after the YAML configuration file to facilitate reproducibility and debugging.

Data Splits and Validation

To assess model generalization, we implemented patient-level cross-validation for each supervised task, using patient IDs as the grouping unit to ensure no data leakage across folds. Each patient appeared exactly once in a test set across all folds, and no overlap occurred between training and test sets. The class distribution within each test set was carefully balanced to include representatives of all clinical or molecular subgroups, ensuring robust evaluation across diverse contexts. Exact patient assignments for each fold are detailed in Supplementary Tables 9.

bioRxiv preprint doi: https://doi.org/10.1101/2025.10.31.685892; this version posted November 3, 2025. The copyright holder for this preprint (which was not certified by peer review) is the author/funder, who has granted bioRxiv a license to display the preprint in perpetuity. It is made available under aCC-BY 4.0 International license.

- **Task 1: LUAD (early-stage / late-stage)**
  Four-fold cross-validation; each fold included approximately 17–18 patients in training and 5–6 patients in testing. Test sets contained both early- and late-stage LUAD patients.

- **Task 2: LUAD (treatment-naïve / TKI)**
  Four-fold cross-validation; each fold had 12–13 patients in training and 4–5 in testing. Test sets included at least one treatment-naïve and multiple TKI-treated patients.

- **Task 3: BRCA (treatment-naïve / anti-PD1)**
  Five-fold cross-validation; each fold contained 49–50 patients in training and 12–13 in testing. Test sets included both pre- and post-treatment patients, typically balanced at ~6 each.

- **Task 4: BRCA (ER^+ / TNBC subtypes)**
  Five-fold cross-validation; each fold included 22–23 patients in training and 5–6 in testing. Test sets included both ER^+ and TNBC patients.

- **Task 5: BRCA (exhaustion status: NE / E)**
  Five-fold cross-validation; each fold included 22–23 patients in training and 6 in testing. Test sets contained both non-exhausted and exhausted samples.

- **Task 6: BRCA (treatment-naïve / chemotherapy)**
  Five-fold cross-validation; each fold included 31–32 patients in training and 7–8 in testing. Test sets included both treatment-naïve and neoadjuvant chemotherapy patients.

bioRxiv preprint doi: https://doi.org/10.1101/2025.10.31.685892; this version posted November 3, 2025. The copyright holder for this preprint (which was not certified by peer review) is the author/funder, who has granted bioRxiv a license to display the preprint in perpetuity. It is made available under aCC-BY 4.0 International license.

					Baseline			GF-V1		GF-V2		GF-V2 [cancer]	GF-V2-Deep	scGPT	scGPT [Cancer]	scFoundation	CellPLM	Scimilarity
Cancer Type	Task-type	Task	Filtration	Cell Type	HVG	scVI	PCA	Zero-shot	Continue	Zero-shot	Tuned	Zero-shot	Zero-shot	Zero-shot	Zero-shot	Zero-shot	Zero-shot	Zero-shot
LUAD	Cancer Stage	Early-stage vs Late stage)	Remove stage IIIB	Cancer Cells	X	X	X	X	-	X	X	X	X	X	X	X	X	X
	Targeted therapy	Treatment naïve vs TKI	Remove metastasis sites	Cancer Cells	X	X	X	X	-	X	X	X	X	X	X	X	X	X
BRCA	Immunotherapy	Treatment naïve vs anti PD1	--	T Cells	X	X	X	X	-	X	X	X	X	X	X	X	X	X
	Clinical subtyping	ER+ vs TNBC	Remove chemo treated. All samples are pre-treatment	Cancer Cells	X	X	X	X	-	X	X	X	X	X	X	X	X	X
	Outcome	Exhausted vs non-exhausted	Remove chemo treated. All samples are pre-treatment	T Cells	X	X	X	X	-	X	X	X	X	X	X	X	X	X
	Chemotherapy	Treatment naïve vs Chemo treated	All samples are pre-PD-1 treatment	Cancer Cells	X	X	X	X	-	X	X	X	X	X	X	X	X	X
BRCA	Cell Annotation	BRCA Cell type (all cells)	All samples are pre-PD-1 treatment	B-cells, T-cells, Fibroblast, Myeloid, pDC, Mast, Endothelial, Cancer	X	X	X	X	X	X		X	X	X	X	X	X	X

Supplementary Table 1 | A matrix of evaluated models (columns) and tasks (rows)

bioRxiv preprint doi: https://doi.org/10.1101/2025.10.31.685892; this version posted November 3, 2025. The copyright holder for this preprint (which was not certified by peer review) is the author/funder, who has granted bioRxiv a license to display the preprint in perpetuity. It is made available under aCC-BY 4.0 International license.

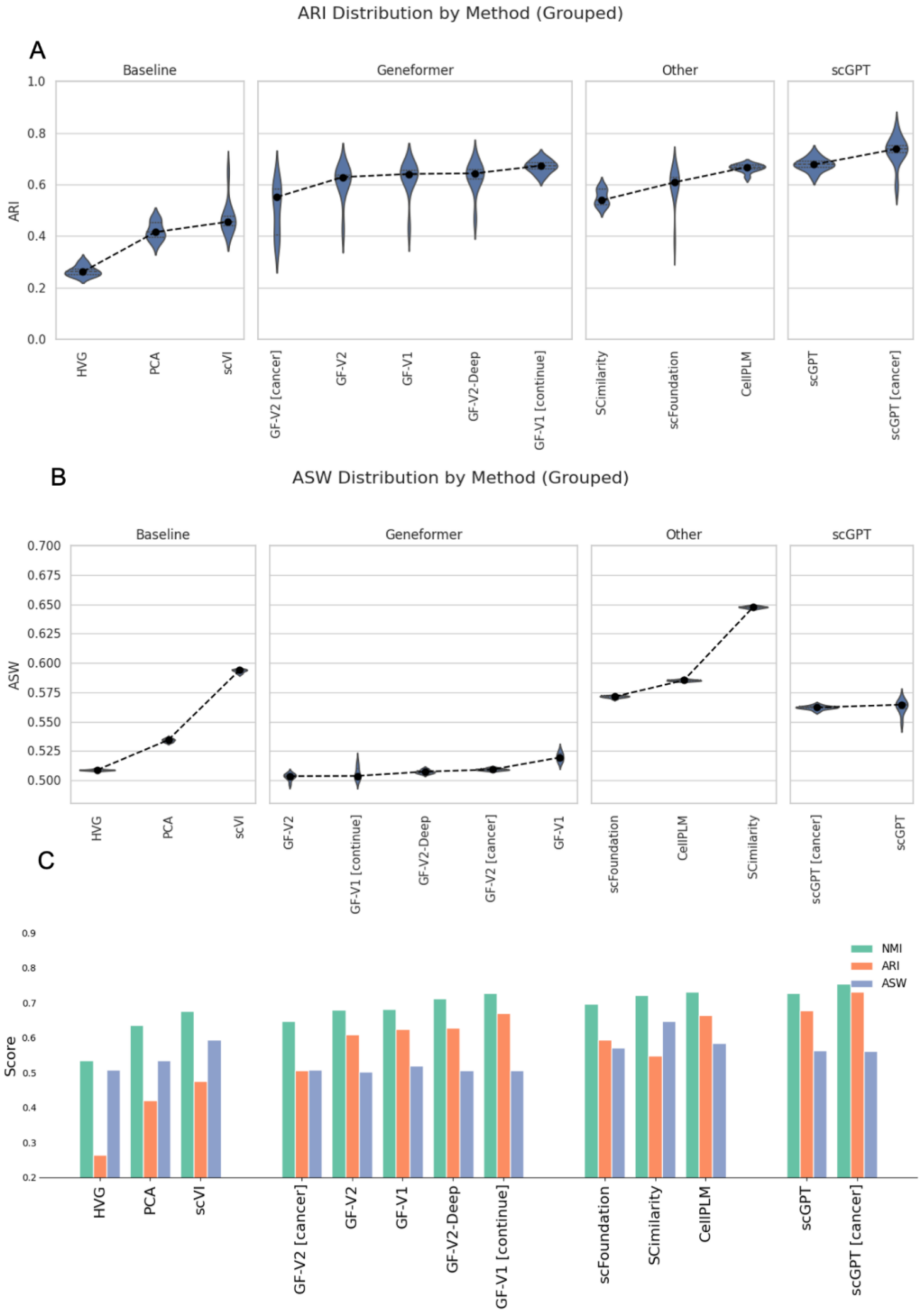

Supplementary Figure 1 | Performance of different models on annotating tumor microenvironment. A) A comparison of the Average Rand Index (ARI) calculated between clusters of the embedding space and the known microenvironment cell types. The embeddings of each model are subsampled by replacement 10 times, and the NMI score for each run is

bioRxiv preprint doi: https://doi.org/10.1101/2025.10.31.685892; this version posted November 3, 2025. The copyright holder for this preprint (which was not certified by peer review) is the author/funder, who has granted bioRxiv a license to display the preprint in perpetuity. It is made available under aCC-BY 4.0 International license.

calculated. B) Average Silhouette Score (ASW) calculated for the model embeddings. Score is normalized between 0 (worst) and 1(best). C) A comparison between the calculated scores (NMI, ARI, and ASW) for all the models.

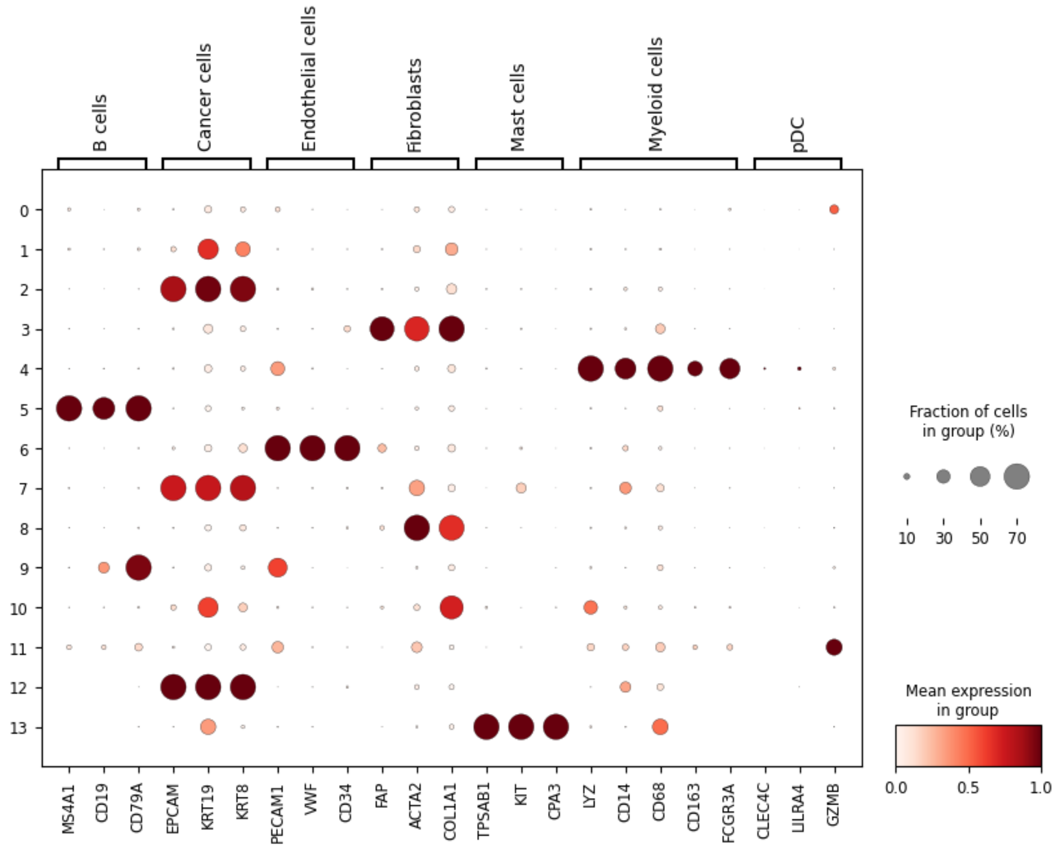

Supplementary Figure 2 | Dot plot visualizing the expression of canonical marker genes across cell clusters identified in the single-cell BRCA data by clustering the embeddings of continually trained GF-V1 [continue]. Each row corresponds to a cell cluster, while each column represents a marker gene grouped by cell type. The size of the dot indicates the fraction of cells within the cluster expressing the gene, and the color intensity reflects the average expression level among those expressing cells.

bioRxiv preprint doi: https://doi.org/10.1101/2025.10.31.685892; this version posted November 3, 2025. The copyright holder for this preprint (which was not certified by peer review) is the author/funder, who has granted bioRxiv a license to display the preprint in perpetuity. It is made available under aCC-BY 4.0 International license.

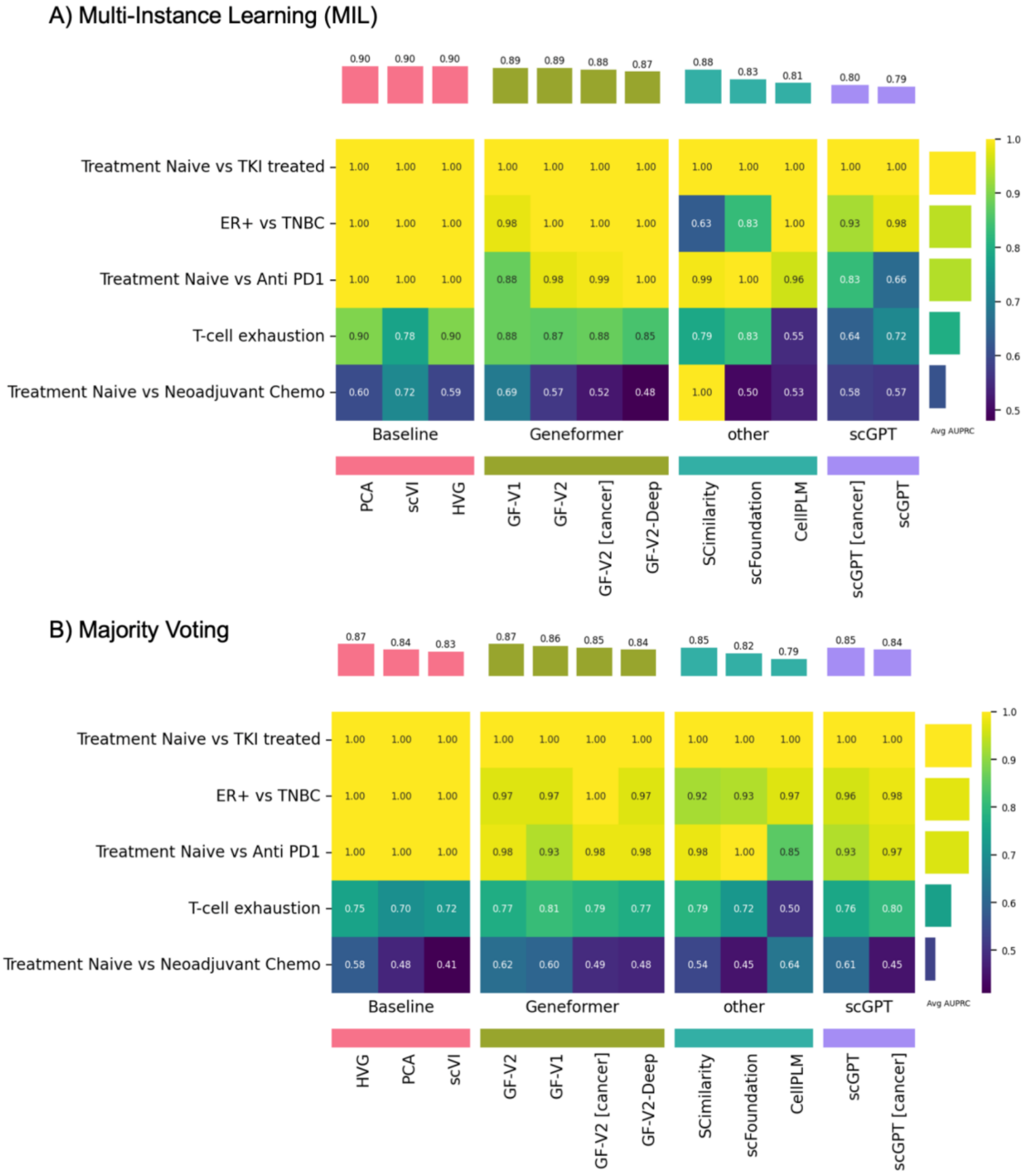

Supplementary Figure 3 | Performance of different models on predicting cancer outcomes. A) Comparison between all models in predicting five different outcomes measured using the area under the precision-recall curve, AUPRC. Each cell represents the average of five cross-validation experiments. The bars on top show the average performance of each model across the different tasks.

bioRxiv preprint doi: https://doi.org/10.1101/2025.10.31.685892; this version posted November 3, 2025. The copyright holder for this preprint (which was not certified by peer review) is the author/funder, who has granted bioRxiv a license to display the preprint in perpetuity. It is made available under aCC-BY 4.0 International license.

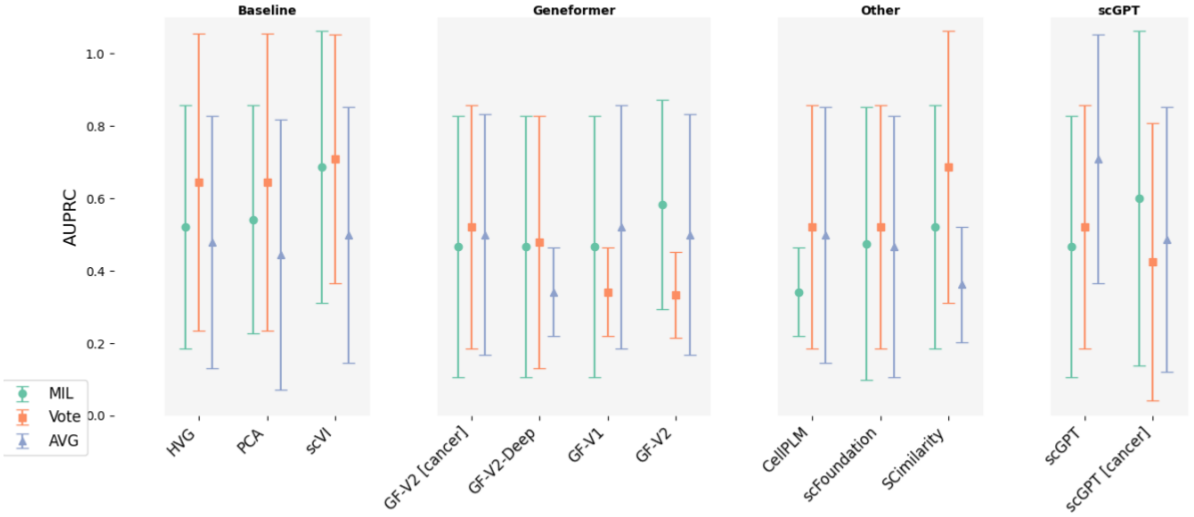

Supplementary Figure 4 | Evaluating the performance of models on predicting cancer stage in lung cancer. All models were evaluated to assess their performance in predicting disease stage (early stage vs. late stage) in lung adenocarcinoma (LUAD) patients. The AUPRC is depicted with this errorbar graph with mean and standard deviation. Most models showed a high level of noise (measured by signal-to-noise ratio (SNR = mean/standard deviation)).

bioRxiv preprint doi: https://doi.org/10.1101/2025.10.31.685892; this version posted November 3, 2025. The copyright holder for this preprint (which was not certified by peer review) is the author/funder, who has granted bioRxiv a license to display the preprint in perpetuity. It is made available under aCC-BY 4.0 International license.

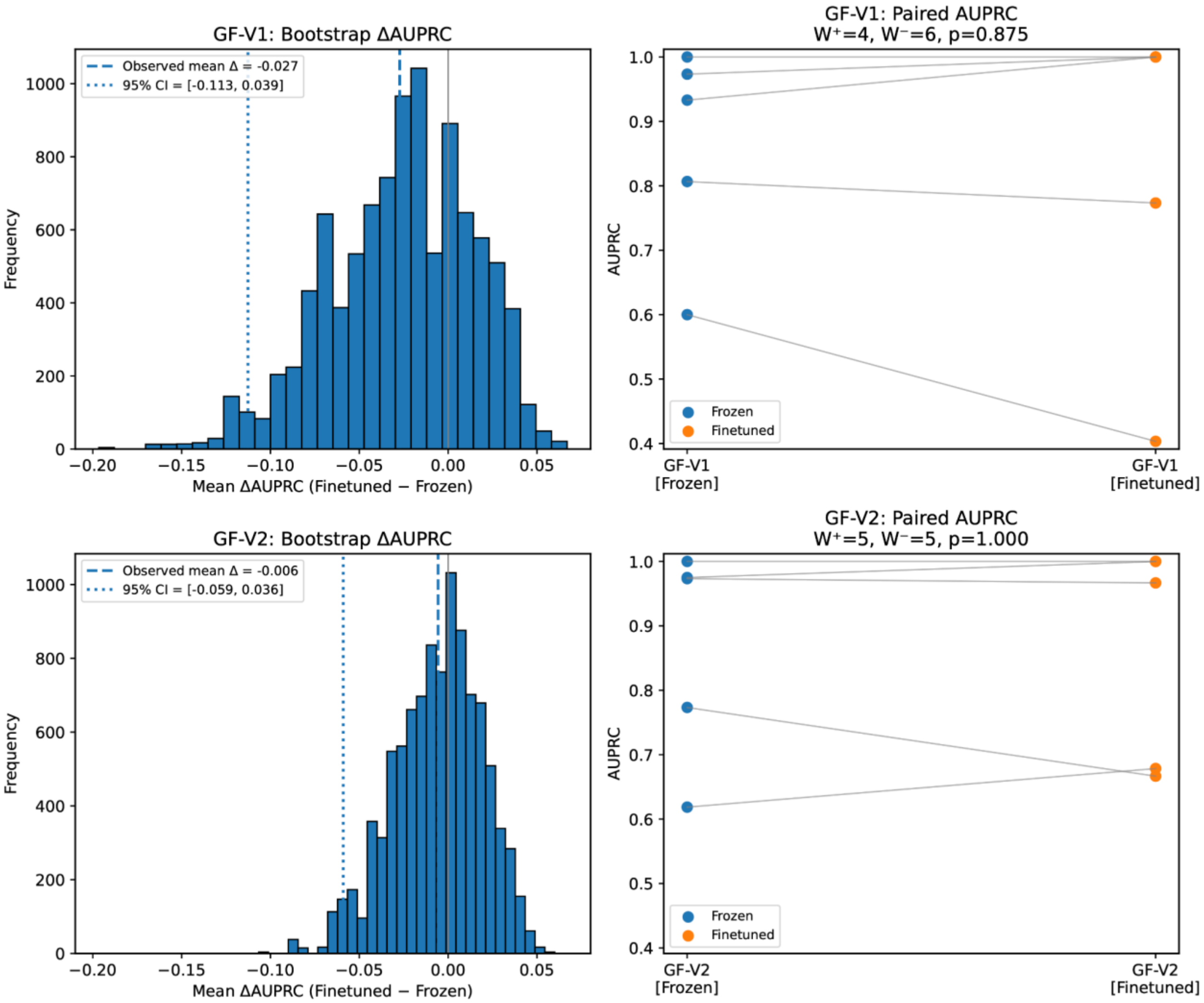

Supplementary Figure 5 | Paired task-level AUPRC comparisons for GF-V2 models. (Top-left) Bootstrap distribution of mean AUPRC differences between GF-V2 (frozen) and GF-V2 [finetuned] across six tasks; vertical lines indicate the observed mean difference and the 95% confidence interval. **(Top-right)** Paired scatter plot of AUPRC values for GF-V2 (frozen) versus GF-V2 [finetuned], with lines connecting the same task to illustrate individual changes. **(Bottom-left)** Bootstrap distribution of mean AUPRC differences between the baseline HVG model and GF-V2 [finetuned] across six tasks; vertical lines indicate the observed mean difference and the 95% confidence interval. **(Bottom-right)** Paired scatter plot of AUPRC values for the baseline HVG model versus GF-V2 [finetuned], with lines connecting the same task.

bioRxiv preprint doi: https://doi.org/10.1101/2025.10.31.685892; this version posted November 3, 2025. The copyright holder for this preprint (which was not certified by peer review) is the author/funder, who has granted bioRxiv a license to display the preprint in perpetuity. It is made available under aCC-BY 4.0 International license.

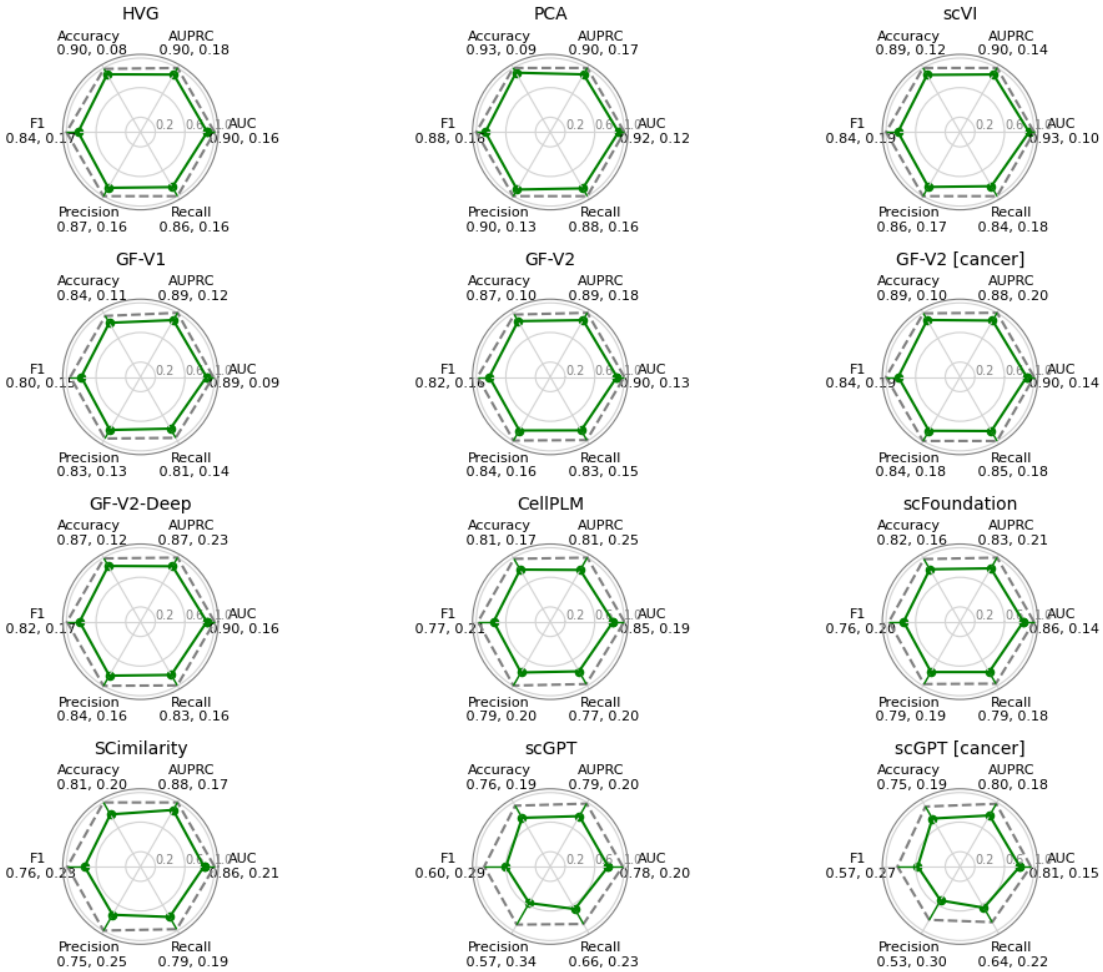

Supplementary Figure 6 | Combined performance of individual models, trained using a multi-instance learning (MIL) framework. The figure comprises nine separate radar charts—arranged in a 3 × 3 grid—corresponding to the embedding methods HVG, PCA, scVI, scGPT, scGPT [cancer], GF-V1, GF-V2, GF-V2 [cancer], and GF-V2-Deep. Each chart has six equally spaced radial axes representing, in clockwise order, Accuracy, AUPRC, AUC, F1, Recall, and Precision. An outer circle marks the unit radius (score = 1), and a corresponding dashed gray polygon indicates the standard deviation. For every model, a solid green polygon connects the mean score of all classification tasks on each metric, and a numeric value pair of the mean score (left) and its standard deviation (right) are written below the score name at the end of each axis.

bioRxiv preprint doi: https://doi.org/10.1101/2025.10.31.685892; this version posted November 3, 2025. The copyright holder for this preprint (which was not certified by peer review) is the author/funder, who has granted bioRxiv a license to display the preprint in perpetuity. It is made available under aCC-BY 4.0 International license.

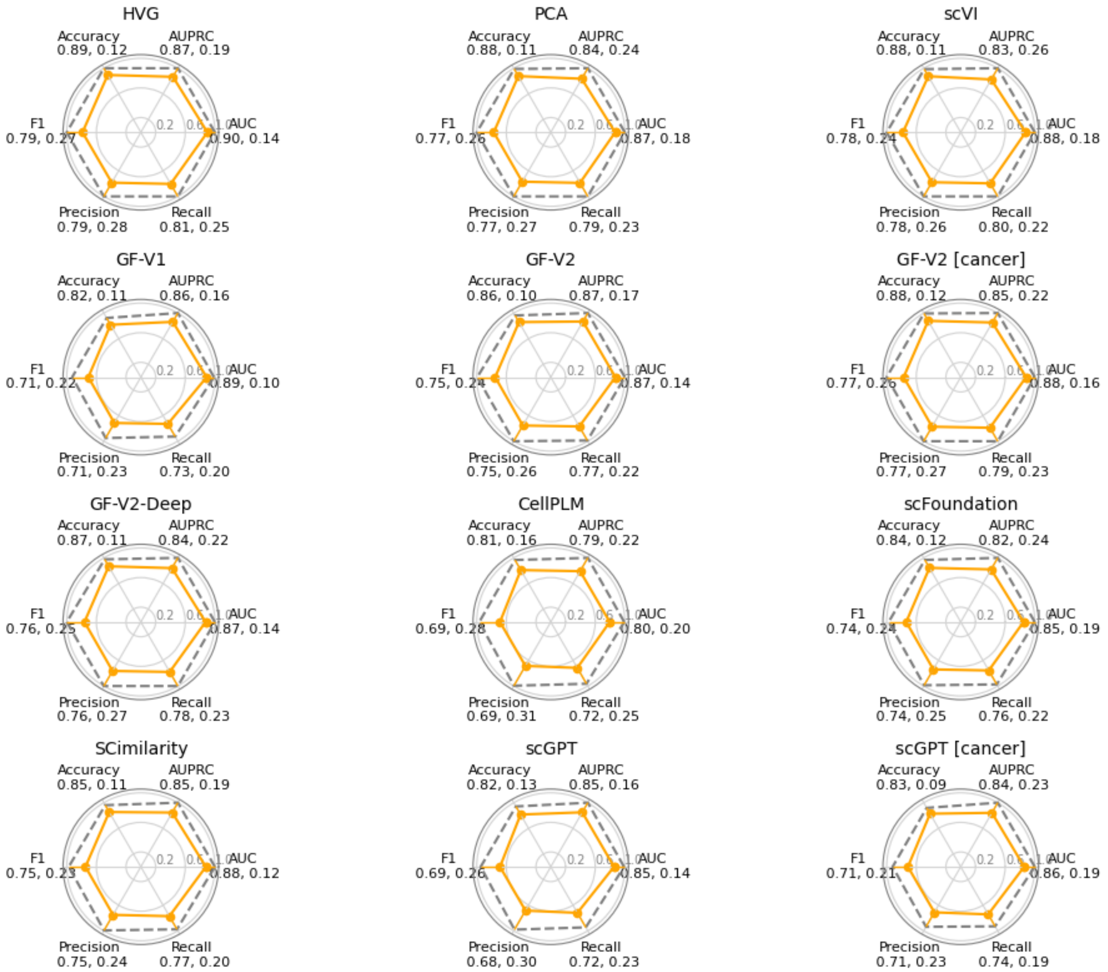

Supplementary Figure 7 | Combined performance of individual models, trained using a majority voting approach. The figure comprises nine separate radar charts—arranged in a 3 × 3 grid—corresponding to the embedding methods HVG, PCA, scVI, scGPT, scGPT [cancer], GF-V1, GF-V2, GF-V2 [cancer], and GF-V2-Deep. Each chart has six equally spaced radial axes representing, in clockwise order, Accuracy, AUPRC, AUC, F1, Recall, and Precision. An outer circle marks the unit radius (score = 1), and a corresponding dashed gray polygon indicates the standard deviation. For every model, a solid orange polygon connects the mean score of all classification tasks on each metric, and a numeric value pair of the mean score (left) and its standard deviation (right) is written below the score name at the end of each axis.

bioRxiv preprint doi: https://doi.org/10.1101/2025.10.31.685892; this version posted November 3, 2025. The copyright holder for this preprint (which was not certified by peer review) is the author/funder, who has granted bioRxiv a license to display the preprint in perpetuity. It is made available under aCC-BY 4.0 International license.

1. Bassez, A. *et al.* A single-cell map of intratumoral changes during anti-PD1 treatment of patients with breast cancer. *Nat. Med.* **27**, 820–832 (2021).
2. Kim, N. *et al.* Single-cell RNA sequencing demonstrates the molecular and cellular reprogramming of metastatic lung adenocarcinoma. *Nat. Commun.* **11**, 2285 (2020).
3. Maynard, A. *et al.* Therapy-induced evolution of human lung cancer revealed by single-cell RNA sequencing. *Cell* **182**, 1232–1251.e22 (2020).
4. Qian, J. *et al.* A pan-cancer blueprint of the heterogeneous tumor microenvironment revealed by single-cell profiling. *Cell Res.* **30**, 745–762 (2020).
